# The clinical diagnosis of symptomatic forefoot neuroma in the general population: a Delphi consensus study

**DOI:** 10.1186/s13047-017-0241-2

**Published:** 2017-12-28

**Authors:** Charlotte Dando, Lindsey Cherry, Lyndon Jones, Catherine Bowen

**Affiliations:** 1 University of Southampton and Solent NHS Trust, Southampton, UK; 2 NIHR Clinical Lecturer in Podiatric Rheumatology, University of Southampton, Southampton and Solent NHS Trust, Southampton, UK; 3 Total Foot Health, Salisbury, UK; 4 University of Southampton, Southampton, UK

**Keywords:** Consensus, Delphi, Forefoot neuroma, Protocol, Diagnosis, Tests

## Abstract

**Background:**

There is limited evidence for defining what specific method or methods should be used to clinically influence clinical decision making for forefoot neuroma. The aim of this study was to develop a clinical assessment protocol that has agreed expert consensus for the clinical diagnosis of forefoot neuroma.

**Methods:**

A four-round Delphi consensus study was completed with 16 expert health professionals from either a clinical or clinical academic background, following completion of a structured literature review. Clinical experience ranged from 5 to 34 years (mean: 19.5 years). Consensus was sought on the optimal methods to achieve the clinical diagnosis of forefoot neuroma. Round 1 sought individual input with an open ended question. This developed a list of recommendations. Round 2 and 3 asked the participants to accept or reject each of the recommendations in the list in relation to the question: “What is the best way to clinically diagnose neuroma in the forefoot?” Votes that were equal to or greater than 60% were accepted into the next round; participant’s votes equal to or less then 20% were excluded. The remaining participant’s votes between 20 to 60% were accepted and placed into the following round for voting. Round 4 asked the participants to rank the list of recommendations according to the strength of recommendation they would give in relation to the question: “What is the best way to clinically diagnose neuroma in the forefoot?” The recruitment and Delphi rounds were conducted through email.

**Results:**

In round 1, the 16 participants identified 68 recommendations for the clinical diagnosis of forefoot neuroma. In round 2, 27 recommendations were accepted, 11 recommendations were rejected and 30 recommendations were assigned to be re-voted on. In round 3, 36 recommendations were accepted, 22 recommendations were rejected and 11 recommendations were assigned to be re-voted on. In round 4, 21 recommendations were selected by the participants to form the expert derived clinical assessment protocol for the clinical diagnosis of forefoot neuroma. From these 21 recommendations, a set of themes were established: location of pain, non weight bearing sensation, weight bearing sensation, observations, tests and imaging.

**Conclusion:**

Following the identification of 21 method recommendations, a core set of clinical diagnostic methods have been prepared as a clinical assessment protocol for the diagnosis of forefoot neuroma. Based on expert opinion, the core set will assist clinicians in forming a clearer diagnosis of forefoot neuroma.

**Electronic supplementary material:**

The online version of this article (10.1186/s13047-017-0241-2) contains supplementary material, which is available to authorized users.

## Background

A neuroma is a benign tumour of the nerve tissue that can develop in various parts of the body [1]. However in the feet it is most commonly associated with the inter metatarsal spaces (IM spaces) of the forefoot [2]. Clinically there are treatment options available although they produce mixed outcomes for relief of symptoms [3]. Contributing to this is the poor understanding of the cause and risks associated with developing forefoot neuroma, although a number of hypothesis have been made [4–7]. Williams and Robinson [8] concluded that there was no single clinical feature that could definitively predict the presence of a forefoot neuroma. Likewise, Owens et al. [9] indicated that there are no pathognomonic diagnostic clinical tests for forefoot neuroma and so clinicians use clinical tests associated with forefoot pathology. As these are non-specific, a clinical diagnosis is achieved through a clinical history and an examination of the foot [10]. Recently, authors have investigated specific clinical tests to determine their sensitivity, specificity, their positive predictive value, and their negative predictive value in clinically diagnosing forefoot neuroma. Mahadevan et al. [11] compared 7 clinical tests to detect forefoot neuroma compared to ultrasound. The overall accuracy rate of these clinical tests were as follows: thumb index finger squeeze (96%), Mulder’s click (61%), foot squeeze (41%), plantar percussion (37%), dorsal percussion (33%), abnormal light touch (26%) and abnormal pin prick (26%). These clinical tests were pre-determined by the authors who recruited from an orthopedic foot and ankle clinic.

Due to the limited statistical evidence documented in publications, the comparisons between the reliability and validity of individualised clinical tests are challenging and thus could potentially explain why identifying a specific test for the clinical diagnosis of forefoot neuroma is difficult. The aim of this study was to develop a clinical protocol that has agreed expert consensus for the clinical diagnosis of forefoot neuroma.

## Method

### Expert identification

The Delphi technique is one example of gaining group consensus in a topic area where evidence is limited or contradictory [1]. Participants who took part in the study were considered experts in the identification of forefoot neuroma. Vernon [1] defined expertise as a ‘variable notion that is determined by the topic for example, clinicians are experts compared to the general population. Therefore it is up to the researcher to state and justify the criteria of an expert for their study’. For this study, the criteria were defined as follows:

#### Inclusion criteria

Experience of diagnosing, assessing or managing at least thirty five cases of neuroma in the forefoot in the last year.

Pathological knowledge of forefoot neuroma.

Individual postgraduate.

Participants who identified themselves as a ‘clinical academic’ highlighted that their workload involved clinical practice as well as academic duties. All of the participants had completed additional training in musculoskeletal health. This included diagnostic imaging modules, masters, PhD’s or consultancy.

### Recruitment

Initially, 10 participants were identified and invited by the researcher (CD) to participate in the study. Identification was determined via a literature review and professional networks. The literature review highlighted authors who had published work in the identification, clinical assessment, or management of forefoot neuroma in the last 50 years. Individuals known by the research team with an interested in musculoskeletal health were contacted to determine their interest in the study, and potentially forward on the study information to their colleagues who met the study criteria. The researcher (CD) invited participants from a number of health professions. Inclusion of a wide range of professions from a range of clinical backgrounds with a geographical diversity is suggested as good practice as it develops the participants to be a representative group [12, 13]. A heterogenous group, with a range of stakeholders, encourages different outlooks and decision-making, which in turn enriches the data leading to better outcomes of credibility and acceptability [14].

The initial 10 participants were then asked to identify and pass on the research information to a further 3 colleagues each who may wish to participate in the study. This sampling technique is known as ‘snowball sampling’ and is particularly effective at identifying individuals in a population who are difficult to contact or have minimal members [15]. This chain referral process continued until a sufficient sample size was reached. Currently there is no consensus of agreement on group size, nor recommendation or unequivocal definition of “small” or “large” [14, 16]. However there is some evidence to suggest that studies with participant groups over 30 rarely produce improved results [17]. In total, 20 participants consented to the study and 16 completed all 4 rounds.

### Ethical approval

Approval for this study was obtained from the University of Southampton, Faculty of Health Sciences, Ethics and Research Governance Online (ERGO) (ID reference: 14,364). All panel members provided electronic or written consent.

### Data collection

A four round Delphi study design was adopted in order to gain a group consensus of opinion via a structured communication process. Consensus was sought on the optimal methods to achieve the clinical diagnosis of forefoot neuroma. An invitation email introduced the topic area with an attached document on the synopsis of present literature/guidance in clinically assessing and diagnosing forefoot neuroma was provided for the participant to read. In addition to this, Round 1 instructions, the Delphi questionnaire and the participant demographic sheet were also attached in the email for the participant to complete. Round 1 sought individual input with an open ended question: “What are your current methods of diagnosing forefoot neuroma?” This developed a list of recommendations. Participants were given a 3 week deadline to complete the questionnaire. At 2 weeks a reminder email was sent to the participant if they had not returned their questionnaire. After the deadline, the questionnaires were collated and duplicated answers were removed and terminology uniformed by the researcher (CD). A ‘comments’ section was part of the questionnaire for participants to elaborate on their thoughts if they felt justification was appropriate. The participants received the whole list and feedback from the first round 2 weeks after the deadline. Rounds 2 and 3 asked the participants to accept or reject each of the recommendations in the list in relation to the question: “What is the best way to clinically diagnose neuroma in the forefoot?” Votes that were equal to or greater than 60% were accepted into the next round; participant’s votes equal to or less than 20% were excluded. The remaining participant’s votes between 20% and 60% were accepted and placed into the following round of voting. The research team (CD, LC, LJ and CB) reviewed previous Delphi publications in healthcare to identify potential threshold values. The literature does not suggest a set agreement scale or rate to define consensus [8]. However there is general concurrence that the researchers should identify and define an agreement scale/rate for consensus to their participants and this is what is adopted for the Delphi study [4, 11]. In round 4, participants were asked to rank the strength of recommendation they would give (where 1 was the lowest rank or lowest strength of recommendation). The top 50% of the responses provided the recommendations for the expert derived clinical assessment protocol. This was determined by the researcher (CD) as an acceptable marker to capture the most valued recommendations for the clinical diagnosis of forefoot neuroma [18]. All participants completed the Delphi through email.

The research team (CD, LC, LJ and CB) made an informed decision to conduct the delphi method through email. From a practical point of view, it allowed the researcher (CD) to converse with participants in a timely manner with minimal interference to the participant’s normal routine. This method of communication allowed a mutual rapport to build and thus increase the likelihood of the participants’ on going commitment to complete the study process [19]. It also meant participants could potentially be accessed globally. An additional benefit included the ability to trace the emails to confirm the participants had received the study information. Most importantly, the participants were anonymised to each other and thus were able to have a voice and share their thoughts on the clinical question without judgement [19].

### Data analysis

#### Descriptive statistics

Nominal demographic data for participants was collected for background information. The data was cleaned and analysed using IBM Statistical Packages for the Social Sciences (SPSS) Version 19.0 for Windows (SPSS Inc., Armonk, NY, USA) to determine: number of cases, mean and standard deviation.

#### Qualitative data

A textual data set was used to capture complex implicit and explicit ideas and phrasing formulated by the delphi question. This body of text was then analysed using thematic analysis to identify and describe the derived themes. This formed the recommendations for the development of the expert derived clinical assessment protocol.

## Results

### Delphi panellist

All 16 participants were based in the United Kingdom. The participant health professional groups were: Podiatrists (*n* = 9), Radiologist (*n* = 1), Rheumatologists (*n* = 2), Orthopeadic surgeons (*n* = 1), Chiropractor (*n* = 1) and Podiatric surgeons (*n* = 2). Clinical experience ranged from 5 years to 34 years (mean 19.5 years) in clinical practice.

### The recommendations

The participants identified 68 recommendations for the clinical diagnosis of forefoot neuroma. Through the Delphi rounds the number of recommendations reduced (Additional files 1 and 2). In total 21 recommendations were finalised. From these 21 recommendations, a set of themes were established: location of pain, non weight bearing sensation, weight bearing sensation, observations, tests and imaging (Table 1).

**Table 1:** The expert derived clinical assessment protocol for the diagnosis of forefoot neuroma

Theme	Delphi Recommendation	Number of Votes (out of 16)
Location of Pain	Pain located in the 2nd or 3rd inter metatarsal space	9
	Forefoot pain reported by patient	7
Patient Reported Symptoms	Paraesthesia radiating distally in the toes.	14
	Pins and needles reported by the patient	12
	Shooting pain reported by the patient	12
	Burning sensations reported by the patient	15
	Clicking reported by the patient	5
Weight Bearing Sensation	Walking on pebbles/lump/stone reported by the patient	9
	Separating the metatarsal heads e.g. met dome, padding, off the shelf insoles ease symptoms	7
	Shoe style: tight fitting/narrow aggravate pain symptoms reported by the patient	10
Observations	On palpation of joint margins no pain reported by the patient	6
	Diastasis of toes	5
	No pain on movement of joint	6
	No swelling	5
Tests	Diagnostic LA (plus/minus steroid injection)	8
	Tenderness/pain reported by patient on palpation of inter metatarsal space (usually 2nd/3rd)	12
	Mulders Click (One hand is clasped around the foot at the level of the metatarsal heads. Whilst compression of the hand around the metatarsal heads the thumb of the contralateral hand exerts firm pressure on the sole of the foot at the site of the suspected forefoot neuroma. The test is considered positive if a palpable click was felt)	15
	Pain reported by patient on lateral compression of the forefoot	11
	Pain on squeezing metatarsal heads (lateral and direct compression)	11
Imaging	Ultrasound	16
	X-ray (rule out other pathology)	11

## Discussion

This study has developed a single clinical protocol, which incorporates 21 recommendations, for the clinical diagnosis of forefoot neuroma. The participants strongly agreed that patient reported symptoms were routinely used to provide a clinical diagnosis. The participants consistently accepted localised forefoot pain and pain specifically reported at the 2nd and 3rd IM spaces to be valuable in aiding the diagnosis of forefoot neuroma. Investigators have extensively discussed the potential aetiology of forefoot neuroma in the 2nd and 3rd IM spaces but little clarity has been found within the literature to determine how valuable “localised forefoot pain” is as an indicator for the diagnosis of forefoot neuroma [2, 5]. Investigators have also reported other patient reported symptoms specifically to the IM spaces such as; paraestheisa, pins and needles, shooting pains and burning sensations [10].

The use of diagnostic ultrasound (US) imaging was the consistently highest scoring recommendation for the clinical diagnosis of forefoot neuroma. Diagnostic US imaging has emerged over the past decade as a useful modality for identification and diagnosis of forefoot neuroma [5, 20], with a number of authors documenting sensitivity and specificity scores of approximately 80 to 95% [21–24]. Thus, there appears to be good agreement between authors on the use of diagnostic US imaging as a reasonable method to be used to differentiate forefoot neuroma from other forefoot pathology. However, the sonographic characteristics for determining the presence of forefoot neuroma were not evaluated as part of this study. Participants just acknowledged that US was an important recommendation for diagnosing forefoot neuroma.

One of the most highly scored tests by the participants was the Mulder’s sign, even though there is evidence showing inconsistency in accuracy of identifying forefoot neuroma through this method [9]. One potential reason for this, is a ‘Mulder’s click’ can be produced with a Mulder’s sign test; it is thought that manipulation of the soft tissue structures or mechanical loading could cause anatomical tissue to bulge or slide over one another creating a false result [25]. However, Mahadevan et al. [11] demonstrated that “squeezing the IM space” produced a tenderness/pain, which had a sensitivity of 96% and a specificity of 100% in 54 ft compared to US findings. Likewise, Owens et al. [9] found 95% of 76 ft had IM space tenderness with the presence of neuroma confirmed by US. Although different terminology is used, both the tests described in the papers by Mahadevan et al. [11] and Owens et al. [9] are identical ‘the symptomatic IM space is squeezed between the tips of the index finger and thumb’. Both investigators also acknowledged the potential use for reproducing pain via lateral compression of the metatarsal heads. Mahadevan et al. [11] demonstrated a 41% sensitivity and 0% specificity in their sample population (*n* = 45 ft) whereas Owen et al. [9], found lateral compression of the metatarsal heads produced a positive response in 88% of their population (*n* = 76 ft). Again little evidence was present in describing pain on compression of the metatarsal heads and what implications this finding has on clinical decision-making.

Most surprisingly, the use of local anaesthesia (LA) (plus or minus a steroid injection) to determine whether the nerves locally in foot are producing the symptoms was also highly indicated. Those participants with the skill set strongly recommended the use of this method, even those who did not have the skill themselves but work in multi-disciplinary team(s) also ranked this highly. Evidence indicates this technique is used to confirm suspicions, if imaging is negative, or for surgical planning [8]. It is appropriate to suggest that this method could potentially be of benefit to those working in teams who have the resources and training to ensure safe, competent practice is achieved.

The participants also commented on the use of X-ray when reported symptoms were poorly defined by the patient and negative clinical tests were documented. Interestingly the use of x-ray was considered an alternative way to exclude other potential pathology and/or for surgical planning [2].

With less certainty, the participants agreed that no swelling, no pain on movement of the metatarsal phalangeal joints (MTPJ’s) and no pain reported on palpation of the joint margins were important to document. These observations are documented in guidelines for assessing joint quality and pathology in patients with MTPJ pain [26]. This suggests that these recommendations were used to rule out other pathologies.

Participants also agreed that patient recall of symptoms in the forefoot was relevant, for example the expression of ‘I’m walking on pebbles, lump or stone’ to describe the sensations in their foot/ft. In some instances, these terms have been used to describe forefoot neuroma [27]. The participants also agreed that the reporting of ‘separating the metatarsal heads’ with either padding or insoles to ease the symptoms was important to understand. Likewise, the participants thought it was important to establish whether a patient’s footwear style aggravated their forefoot symptoms. The NICE guidelines for neuroma advise that individuals who chose to wear narrow or tight fitting footwear, usually with a heel, often report that their footwear aggravates their symptoms therefore health professionals should advise patients to modify their footwear (broader shoe style) [28, 29].

### Study limitations

This study design was able to assimilate current methods used for the clinical diagnosis of forefoot neuroma. However the Delphi design has never been proved or disproved to significantly improve judgment in identifying or forecasting specific topic issues in healthcare, information technology or business, thus there is potential for the recommendations to not be precise [30]. There is an assumption that agreement between the participants would reduce the risk of outcomes being invalid [31]. One way in which the reliability of the recommendations were reviewed, was for the researcher (CD) to feedback the developing opinions at group level. It was anticipated that this would encourage disagreements or concerns to be raised. Hasson et al. [31] proposed the idea that if the panel members were not able to reflect or elaborate on their answers then this could be potentially seen as forced consensus. Therefore a section for “comments” was available for panel members to elaborate on their thoughts. Hasson et al. [31] also proposed that recommendations are strengthened when opinions are challenged anonymously, thus increasing validity. The ‘comments’ section provided insight and reflection for the researcher (CD) to check each panel members’ meaning, accuracy and consistency of a phrase throughout all 3 rounds.

Another potential study limitation could have been the participant sample recruited. The study was accessing participants who have a pre-existing interest in the topic, which in turn would increase content validity but could be affected by the response rate [31]. There is a risk that those invested in the study may modify their opinions to fit with the majority or with current clinical practice. To reduce this, Hsu and Sandford [32] advise a qualitative and quantitative element to the Delphi design in order to understand the priorities within the topic area. For the clinical diagnosis of forefoot neuroma, the ranking of recommendations allowed the panel members to vote for specific methods to identify this condition rather than a holistic approach.

### Future recommendations

Based on the findings from this study, any proposed future research on the diagnosis of forefoot neuroma should consider validating the recommendations in supporting clinical decision-making in clinical practice. Also, there is a need to develop and test a diagnostic scoring system based on the identified recommendations from this study to diagnose forefoot neuroma.

## Conclusion

Following the identification of 21 method recommendations, a core set of clinical diagnostic methods have been prepared as a clinical assessment protocol for the diagnosis of forefoot neuroma. Based on expert opinion, the core set will assist clinicians in developing a clearer diagnosis of forefoot neuroma.

## Additional files

Additional file 1: Round 2 votes of the accepted, rejected and re-voted methods for the clinical diagnosis of forefoot neuroma. (DOCX 12 kb)

Additional file 2: Round 3 votes of the accepted, rejected and re-voted methods for the clinical diagnosis of forefoot neuroma. (DOCX 13 kb)

## Abbreviations

ERGO: Ethics and Research Governance Online
IM space: Inter metatarsal space
LA: Local anesthesia
MTPJ: Metatarsal phalangeal joint
SPSS: Statistical Packages for the Social Sciences
US: Ultrasound
