# Supplementary material for: The clinical diagnosis of symptomatic forefoot neuroma in the general population: a Delphi consensus study
Source: J Foot Ankle Res. 2017 Dec 28;10:59. doi: 10.1186/s13047-017-0241-2 (PMC5745595; doi:10.1186/s13047-017-0241-2)
Supplement: Supplementary file 1 — Round 2 votes of the accepted, rejected and re-voted methods for the clinical diagnosis of forefoot neuroma. (DOCX 12 kb) [file 13047_2017_241_MOESM1_ESM.docx]

**Additional File 1**

Round 2 votes of the accepted, rejected and re-voted methods for the clinical diagnosis of forefoot neuroma.

| **Accepted** | Patient reported the pain is sporadic | Lack of other pathology or differential diagnosis | Checking for nerve impingement |
| --- | --- | --- | --- |
| Weight bearing activity aggravates symptoms | Footwear removed relieves pain symptoms | Pain after weight bearing activity | Shoe style: tight fitting/narrow aggravates pain symptoms |
| Ultrasound (also used to confirm diagnosis) | MRI | X-ray (rule out other pathology/surgical planning) | Rule out radiculopathy/symptoms |
| Rule out MTPJ pathology | Mulders click/sign (not always present) | Pain on squeezing the metatarsal heads (lateral and direct compression) | Paraesthesia radiating distally in the toe(s) |
| Patient reports walking on pebbles/marble or stone | Tenderness/pain on palpation of the inter metatarsal space (usually 2^nd^/3^rd^) | Pain on lateral compression of the forefoot | Pain in between the metatarsal heads and no directly upon them. |
| Patient reports tingling | Patient reports a shooting sensation | Patient reports a numbness | Pain extending to the toe(s) |
| Patient reports pins and needles | Patient reports a burning sensation | Patient reports electric shock(s) (feeling) | Abnormal sensation In the toe(s) |

| **Re-voted** |  | Patient reports forefoot pain | Tightness or reduced space in the inter metatarsal space |
| --- | --- | --- | --- |
| Clicking reported by the patient | Cramps reported by the patient | Patient reports a sharp pain | No pain on movement of the MTPJ |
| Pain able to create the pain (yes + no) | Checking for constant or intermittent pain | Slightly vague or nebulous description of the pain and location | Visual Analogue scale |
| No heat/redness | Skin and tissue should look normal | Medication checked | Monofilament and peripheral sensation checked |
| Forefoot deformity | Diastasis of toes | Joint margins palpated: no pain reported | Light bulb effect: pain switching on and off |
| Separating metatarsal heads relieves symptoms | No swelling | Pulses normal with no warmth to the joint | Rule out tarsal tunnel |
| Pain located in the 2^nd^/3^rd^ inter metatarsal space | Previous treatments failed | Undertaking new activities increases symptoms. | Diagnostic LA (plus or minus steroid injection) |
| Co-morbidities checked | Biomechanical alteration/difference to foot/ankle | General aggravating factors are established | General relieving factors are established |
