# Supplementary material for: The clinical diagnosis of symptomatic forefoot neuroma in the general population: a Delphi consensus study
Source: J Foot Ankle Res. 2017 Dec 28;10:59. doi: 10.1186/s13047-017-0241-2 (PMC5745595; doi:10.1186/s13047-017-0241-2)
Supplement: Supplementary file 2 — Round 3 votes of the accepted, rejected and re-voted methods for the clinical diagnosis of forefoot neuroma. (DOCX 13 kb) [file 13047_2017_241_MOESM2_ESM.docx]

**Additional file 2**

Round 3 votes of the accepted, rejected and re-voted methods for the clinical diagnosis of forefoot neuroma.

| **Accepted** | Ultrasound (also used to confirm diagnosis) | Patient reports a burning sensation | Mulders click/sign (not always present) |
| --- | --- | --- | --- |
| Paraesthesia radiating distally in the toe(s) | Pain in between the metatarsal heads and no directly upon them. | Patient reports pins and needles | Rule out radiculopathy/symptoms |
| Patient reports a shooting sensation | Tenderness/pain on palpation of the inter metatarsal space (usually 2^nd^/3^rd^) | Pain on lateral compression of the forefoot | Pain on squeezing the metatarsal heads (lateral and direct compression) |
| X-ray (rule out other pathology/surgical planning) | Lack of other pathology or differential diagnosis | MRI | Pain after weight bearing activity |
| Patient reports forefoot pain | Undertaking new activities increases symptoms. | No heat/redness | Checking for constant or intermittent pain |
| Diagnostic LA (plus or minus steroid injection) | Previous treatments failed | Patient reported pain is sporadic | Patient reports a sharp pain |
| Separating metatarsal heads relieves symptoms | Cramps reported by the patient | Skin and tissue should look normal | Joint margins palpated: no pain reported |
| No pain on movement of the MTPJ | Clicking reported by the patient | Diastasis of toes | Shoe style: tight fitting/narrow aggravates pain symptoms |
| Pain able to create the pain (yes + no) | Pain located in the 2^nd^/3^rd^ inter metatarsal space | Patient reports walking on pebbles/marble or stone | Pain extending to the toe(s) |
| No swelling |  |  |  |

| **Re-voted** |  | Patient reports a numbness | Abnormal sensation In the toe(s) |
| --- | --- | --- | --- |
| Footwear removed relieves pain symptoms | Patient reports tingling | Weight bearing activity aggravates symptoms | Rule out MTPJ pathology |
| Checking for nerve impingement | Patient reports electric shock(s) (feeling) | General relieving factors are established | General aggravating factors are established |

| **Excluded** | Co-morbidities checked | Rule out tarsal tunnel | Light bulb effect: pain switching on and off |
| --- | --- | --- | --- |
| Medication checked | Monofilament and peripheral sensation checked | Pulses normal with no warmth to the joint | Slightly vague or nebulous description of the pain and location |
| Tightness or reduced space in the inter metatarsal space | Visual Analogue scale | Biomechanical alteration/difference to foot/ankle | Pain in the lateral forefoot area |
| Patient reports a popping sensation | No previous trauma or injury | No joint instability | Forefoot deformity |
| No pain on pressing the plantar forefoot region | Normal foot shape | Temperature checked | Reduced mobility of MTPJ |
| Patient ‘unable to place a finger on it’ | Joint stiffness in the MTPJ | Patient reports a ‘dislocating sensation of the toes’ |  |
